# Computer Aided Diagnosis System for Detection of Cancer Cells on Cytological Pleural Effusion Images

**DOI:** 10.1155/2018/6456724

**Published:** 2018-11-08

**Authors:** Khin Yadanar Win, Somsak Choomchuay, Kazuhiko Hamamoto, Manasanan Raveesunthornkiat, Likit Rangsirattanakul, Suriya Pongsawat

**Affiliations:** ^1^Faculty of Engineering, King Mongkut's Institute of Technology Ladkrabang, Bangkok, Thailand; ^2^School of Information and Telecommunication Engineering, Tokai University, Tokyo, Japan; ^3^Department of Pathology, Faculty of Medicine, Srinakharinwirot University, Nakhon Nayok, Thailand

## Abstract

Cytological screening plays a vital role in the diagnosis of cancer from the microscope slides of pleural effusion specimens. However, this manual screening method is subjective and time-intensive and it suffers from inter- and intra-observer variations. In this study, we propose a novel Computer Aided Diagnosis (CAD) system for the detection of cancer cells in cytological pleural effusion (CPE) images. Firstly, intensity adjustment and median filtering methods were applied to improve image quality. Cell nuclei were extracted through a hybrid segmentation method based on the fusion of Simple Linear Iterative Clustering (SLIC) superpixels and K-Means clustering. A series of morphological operations were utilized to correct segmented nuclei boundaries and eliminate any false findings. A combination of shape analysis and contour concavity analysis was carried out to detect and split any overlapped nuclei into individual ones. After the cell nuclei were accurately delineated, we extracted 14 morphometric features, 6 colorimetric features, and 181 texture features from each nucleus. The texture features were derived from a combination of color components based first order statistics, gray level cooccurrence matrix and gray level run-length matrix. A novel hybrid feature selection method based on simulated annealing combined with an artificial neural network (SA-ANN) was developed to select the most discriminant and biologically interpretable features. An ensemble classifier of bagged decision trees was utilized as the classification model for differentiating cells into either benign or malignant using the selected features. The experiment was carried out on 125 CPE images containing more than 10500 cells. The proposed method achieved sensitivity of 87.97%, specificity of 99.40%, accuracy of 98.70%, and F-score of 87.79%.

## 1. Introduction

**Figure 1:**
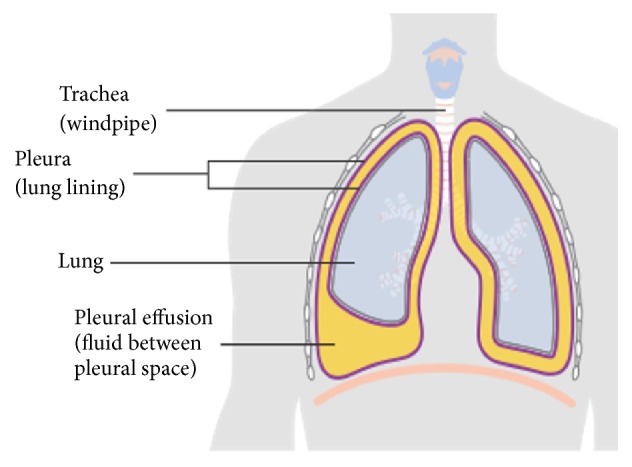
The presence of pleural effusion in the pleural cavity [2].

Pleural effusion or pulmonary effusion (PE) is the pathologic accumulation of fluid in the pleural cavity, between the visceral and parietal layers surrounding the lung, as demonstrated in Figure 1 [1, 2]. Normally, the pleural space is lined by a thin layer of mesothelial cells and contains about 5-10 ml of clear fluid for lubrication during respiratory movement. When cancer cells grow or spread to the pleura, they cause malignant pleural effusion (MPE). Half of all cancer patients have a high possibility of developing MPE. Both primary and metastatic cancers can lead to a diagnosis of MPE. Mesothelioma, a rare form of cancer, is the primary cancer of the pleura. Lung cancer and breast cancer are the most frequent metastatic cancers in male and female patients, respectively. Both malignancies are responsible for about 50-65% of MPE. Lymphoma, tumors of the genitourinary tract, and gastrointestinal tract are responsible for 25%. Tumors of unknown primary account for 7-15% of all MPE [3]. From statistics, as mentioned earlier, MPE is mostly caused by the invasion of metastatic cancer to the pleura. Metastatic cancer is the major cause of cancer morbidity and mortality. It is estimated that metastasis is responsible for about 90% of cancer deaths. Although cancer in the pleural effusion is seen in advanced stages of malignancy and leads to rapid mortality, the survival time can be prolonged by earlier diagnosis together with prompt and effective treatment to slow cancer progress. Currently available tools for detecting the presence of MPE in the pleura are cytology, cytometry, and imaging modalities such as X-ray, Ultrasound, Computed Tomography (CT), and Magnetic Resonance Imaging (MRI). For the assessment of malignancy, cytological examinations are widely used by pathologists because they are simple, cheap, less invasive, and highly useful tools [4].

In a cytological examination, fluid from the malignant pleural effusion is collected and smeared on cytological glass slides using the staining methods. Then, cytologists or pathologists visually examine for morphology changes and visual abnormalities in every single cell under a microscope to determine malignancy prevalence [5]. Manual screening of cytology slides is tedious and subjective to inter- and intra-observer bias. Since the presence of MPE implies advanced malignancy and reduced survival, it is crucial to diagnose malignancy in MPE as early and speedy as possible. Thanks to recent improvements in medical technology, automated image analysis has the potential to allow for earlier and faster diagnosis with more accurate and objective diagnosis results. Hence, reliable CAD systems using CPE images are in high demand. They can serve as an essential tool to assist cytologists in the assessment of malignancy; however, complex and unusual cases still require further examination by cytologists. The benefits of CAD systems are that they accelerate the diagnosis process, make diagnosis objective, and reduce any diagnostic divergence resulting from different observers. Consequently, they allow for the early and speedy diagnosis and prognosis of cancer cells and help oncologists in making effective treatment plans promptly.

Few researchers have researched the analysis of CPE images for the automatic detection of cancerous cells from CPE specimens. In 2001, F. Chen et al. [6] proposed the automated classification of adenocarcinoma and healthy cells (especially mesothelial cells and lymphocytes) in CPE images. Morphology and wavelet features were used as inputs to a backpropagation neural network to discriminate between adenocarcinoma and benign cells. Their study was based on 60 adenocarcinoma cells and many (the number was not specified numerically) benign cells. Unfortunately, the authors did not provide a method for segmenting nuclei nor an evaluation of classification performance. L. Zhang et al. 2006 [7] introduced a fuzzy recognition method to classify four types of cells, namely, healthy cells, cancer cells, mild dyskaryotic cells, and severe dyskaryotic cells. Otsu thresholding and fuzzy edge detection were used to segment the cells. Seven morphological features were extracted from each segmented cell and fed as input into a fuzzy recognition system to classify those four types of cells. However, there was a lack of clarity in the evaluation process in [4, 5]. This has encumbered the reproduction of these methods for practical use. A.B. Tosun et al. 2015 [8] presented the automated detection of malignant mesothelioma using nuclei chromatic distribution. Firstly, the nuclei were extracted using a semiautomatic approach in which the initial contour of cell nuclei was manually segmented under the guidance of cytologists, and level set method was utilized to finalize the contour of cell nuclei. For each extracted nucleus, its linear optimal transport (LOT) was computed and subjected to linear discriminant analysis based on k-nearest neighborhood algorithm classifier to differentiate between mesothelioma and benign cells. Their experiment was based on 1080 cell nuclei containing 590 mesotheliomas and 490 benign nuclei and obtained 100% accuracy. Unfortunately, their method was not fully automated since cell segmentation was manually performed. Moreover, none of the methods mentioned above deals with the overlapped cell problem. Decomposing overlapped cells into their constituents would enhance analysis performance and robustness. As such, the approaches mentioned thus far focus on detecting specific types of cancer cells such as adenocarcinoma or mesothelioma cells in CPE images. Meanwhile, an early and essential task in clinical practice is to differentiate between benign cells and cancer cells regardless of specific cancer types. This may then be followed by classifying cancer cells into the different types (i.e., lung carcinoma, mesothelioma, breast carcinoma, and so on). In practice, a tool that can detect malignant cells from all MPE cases is in high demand. Despite being linked with high rates of morbidity and mortality, research efforts for the automated analysis of MPE are still limited compared to other areas such as cervical cancer, breast cancer, lung cancer, and so on. Thus, automated analysis of pleural effusion samples remains to be widely researched.

To advance the utilization of MPE analysis, we propose a novel CAD system based on the analysis of CPE images which can classify cells as either benign or malignant. The main distinction of the proposed method from previous literature is that it can detect malignancy in all MPE cases. Our newly designed system is a fully automated system that addresses the overlapped cell and unbalanced-data problems which have so far been left unsolved. In addition, the proposed method takes advantage of the selection of dominant features using a hybrid metaheuristic method. Our system includes seven main stages: preprocessing, cell nuclei segmentation, postprocessing, overlapped cell nuclei isolation, feature extraction, feature selection, and classification. The preprocessing stage aims to improve the quality of the images. In the segmentation stage, our developed hybrid superpixel-driven K-Means clustering method, known as SLIC/K-Means hybrid, was used to extract cell nuclei regions. Then, a series of morphological operations were employed to improve segmented cell nuclei boundaries and eliminate any false findings. Subsequently, the combination of the shape-based analysis and concavity analysis was applied to isolate any overlapping nuclei into individual ones. After the cell nuclei were segmented, a total of 201 features from morphometric, colorimetric, and textural features were extracted to create an initial feature set. Our novel hybrid SA-ANN feature selection approach was employed to obtain the optimal feature set that encompasses the most discerning features. The optimal feature set was fed as input to an ensemble classifier of bagged decision trees to classify benign and malignant cells.

This paper is divided into five sections. In this section we have presented an introduction to the diagnosis of malignancy in PE and outlined related works. The description of the studied dataset is given in Section 2.  Section 3 describes the methodology used by the proposed CAD system.  Section 4 discusses the experimental results.  Section 5 concludes and presents the scope for future work.

## 2. Dataset Description

To date, there is no publicly available dataset of CPE images. Thus, we prepared the local dataset through the cooperation with experts from the Department of Pathology, Faculty of Medicine, Srinakharinwirot University, Thailand. The local dataset is based on the microscope images captured from the archival cytology glass slides of pleural effusion samples from the university mentioned earlier. Firstly, all samples were stained on the glass slides with a classical Papanicolaou (Pap) staining method which can provide good cellular morphology when inspected by the optical microscope [9, 10]. Then, two skilled and certified cytologists captured the digitized cytology images from the glass slides through a digital camera mounted to a light microscope with 40x magnification. Thereafter, they analyzed every single cell within the collected images and annotated the regions of the interest (i.e., cancer cells), which were used as the ground truth. The dataset with associated ground truth consists of 125 CPE images containing benign and malignant cells. The images have resolutions of 4050 x 2050 pixels and 4080 x 3702 pixels and are stored in 8-bit RGB space.

## 3. Methodology

**Figure 2:**
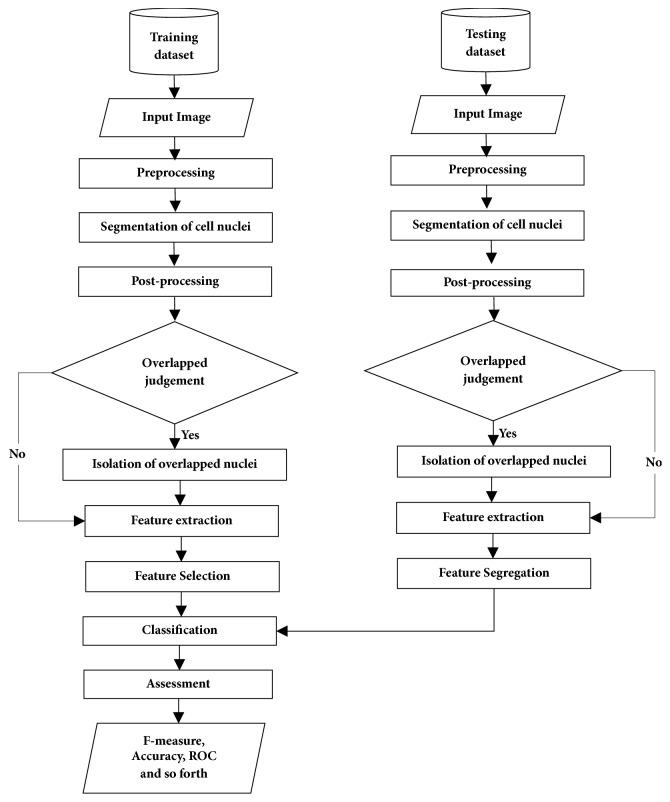
System framework of the proposed CAD system.

The framework of the proposed CAD system is presented in Figure 2. The method involves seven major stages: (a) preprocessing, (b) nuclei segmentation, (c) postprocessing, (d) identification and isolation of overlapped cell nuclei, (e) feature extraction, (f) feature selection, and (g) classification.

### 3.1. Preprocessing Stage

**Figure 3:**
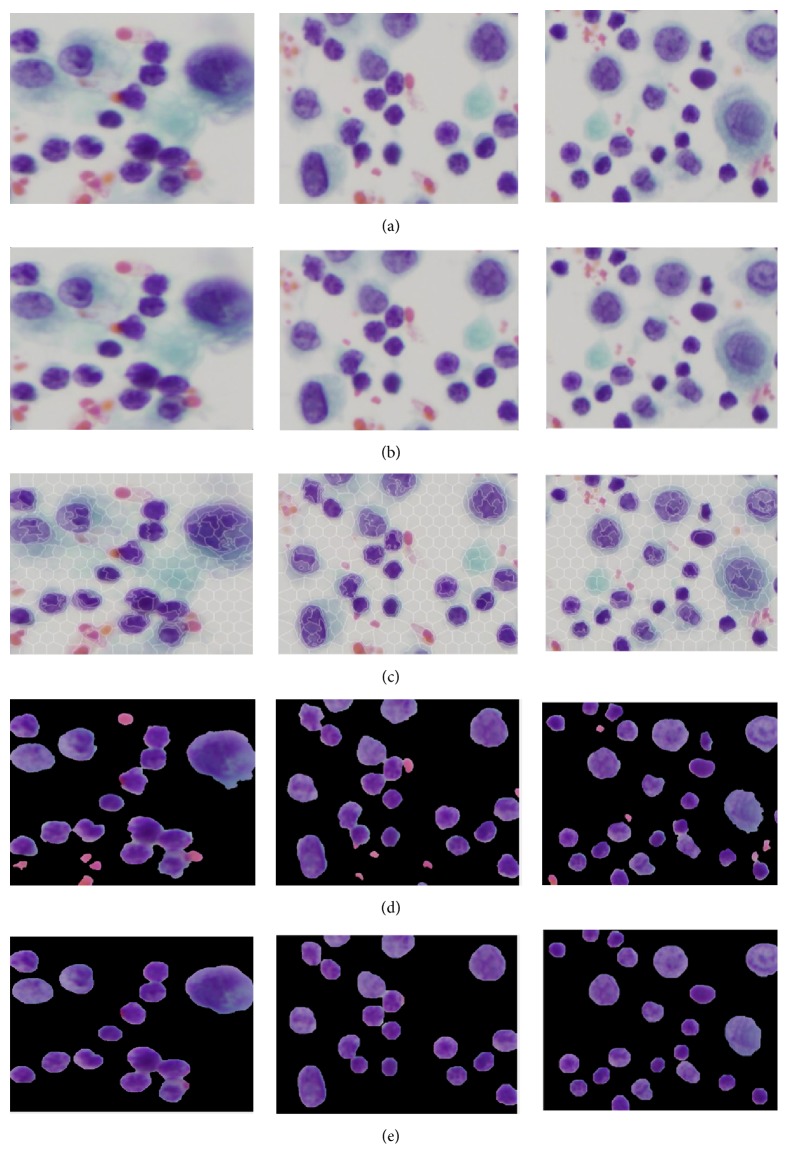
Visual results of segmenting cell nuclei from CPE images: (a) original image, (b) preprocessed image, (c) superpixels segmentation using SLIC, (d) K-Means based unsupervised color segmentation on SLIC superpixels, and (e) postprocessed image (refinement of nuclei boundary and elimination of false findings).

During the staining of PE samples and digitalizing of CPE images, there is usually a degradation in quality, which includes uneven staining, uneven lighting, poor contrast, and the presence of additive noise. Therefore, preprocessing is essential in dealing with image quality prior to the main analysis. Firstly, the images were resized into 1024 x 1024 pixels in order to achieve image normalization, standardization, and computation time reduction. Then, each image was enhanced using an image intensity adjustment method that increases the contrast between the foreground (region of interests) and background [11]. In order to reduce noise without losing cell-edge clarity, R, G, and B components were separated from the original RGB image. Then, a median filter [12] was applied to each color component independently. Finally, the filtered RGB image was obtained by combining the filtered R, G, and B components together. The visual results before and after applying preprocessing to different images are depicted in Figures 3(a) and 3(b).

### 3.2. Segmentation of Cell Nuclei Using a Novel Hybrid SLIC/K-Means Algorithm

Segmentation is one of the most essential processes in biomedical image analysis. Most of the image analysis in cytology and histology is focused on nuclei segmentation since cell nuclei provide more significant diagnostic value than other cell parts. To determine cell malignancy, the cell nucleus needs to be segmented from the background (i.e., cytoplasm, red blood cells). Then, malignancy is predicted based on certain features extracted from each nucleus. Since the results of nuclei segmentation have a high impact on all subsequent analysis, it is crucial that the nuclei are accurately extracted.

Few researchers have studied the automated segmentation of cells or nuclei in CPE images. E. Baykal et al. 2017 [13] introduced an active appearance model to segment nuclei from the background in CPE images and compared it with color thresholding, clustering, and graph-based methods. They obtained 98.77% accuracy. However, their approach was designed to segment an image with only one cell. It is hard to use this in practice since there may be up to a million cells in one image. In [14], they investigated the detection of cell nuclei using supervised learning approach. The approach is based on the combination of Haar filter and AdaBoost classifier. Three images with a total of 178 nuclei were used for testing. A True Positive Rate of 89.32% and False Positive Rate of 5.05% were obtained. Their framework performed well with an independent cell nucleus; however, it showed limitations when it came to segmenting overlapped cell nuclei. Moreover, it required extensive prior knowledge to train the classifier. In our previous works [15], we have proposed several alternative nuclei segmentation methods such as Otsu thresholding approach, K-Means clustering approach [16], and supervised pixel classification using ANN [17] on a small dataset (24 CPE images). Recently, we collected more images and built a new dataset containing 35 CPE images. Using that new dataset, we employed twelve segmentation methods: (1) the Otsu method, (2) an ISODATA thresholding method, (3) a maximum entropy thresholding method, (4) cross-entropy thresholding, (5) minimum error thresholding, (6) fuzzy entropy thresholding, (7) adaptive thresholding, (8) K-Means clustering, (9) fuzzy C-means clustering, (10) mean shift clustering, (11) Chan-Vese level set, and (12) graph cut methods to extract the cell nuclei from CPE images, and we compared the results attained [18]. From the comparison results, Otsu, K-Means, mean shift clustering, graph cut method, and a Chan-Vese level set method provided promising segmentation results. Although Otsu provided promising results with low computational time, the segmentation accuracy of Otsu showed degradation in images with a high level of noise because Otsu is sensitive to noise. The images in the studied dataset (124 images) have a great deal of noise. K-Means, mean shift, Chan-Vese, and graph cut methods were found to be computationally expensive especially with images containing a high population of cells. For machine learning based segmentation methods, prior knowledge is required to train a learning model. Thus, there are still opportunities for further enhancements in the nuclei segmentation of CPE images. Reliable nuclei segmentation stays challenging due to the high population of cells and high diversity of cell appearance. In this study, we present a hybrid novel SLIC/K-Means based nuclei segmentation method in which SLIC superpixels are used as a presegmentation step to minimize the computational time of K- means clustering.

The first step of the hybrid SLIC/K-Means method is to perform superpixel segmentation as a presegmentation step. Superpixels fragment the image into a set of structurally meaningful segments where the boundaries of each segment take into the consideration the edge information from the original image. Superpixels are used in the preprocessing stage for object recognition and medical image segmentation. Among the various superpixel segmentation techniques, we opted for a SLIC algorithm because SLIC generates compact superpixels with a more regular shape (R. Achanta et al. [19]). By breaking the image into regularly shaped superpixels, it is easier to distinguish between the nuclei and background depending on the superpixel shape. Moreover, SLIC is simple to implement. It requires only the number of desired superpixels as the input parameter and needs a low computation time compared to other superpixel techniques [20]. SLIC generates compact, uniform superpixels by clustering pixels based on their color similarity and proximity. This is done by using a combined 5-dimensional space [labxy], where l, a, b constitute the pixel color vector in LAB color model and xy denotes the x and y positional coordinates of the pixel position (x, y coordinates). SLIC takes as input the desired number of approximately uniform superpixels. Once SLIC generated the superpixels, we determined the median color feature of each superpixel region in the L*∗*a*∗*b*∗* color space. K-Means clustering [21] was then utilized to classify the color feature of each compact superpixel into nuclei or non-nuclei, rather than having to perform clustering over the full original image pixels. Since representing the image as SLIC superpixels can give more accurate boundary information than representing the image by pixels, performing presegmentation using SLIC superpixels before K-Means clustering allows us to preserve the natural shape of cell nuclei. Also, it can reduce the complexity of the algorithm dramatically. This happens because the number of superpixels is much smaller than the number of pixels. Hence, applying K-Means clustering on SLIC superpixels, rather than on pixels, can improve the algorithm efficiency and lead to rapid computation. The visual results of nuclei segmentation on different images are illustrated in Figures 3(c) and 3(d).

### 3.3. Postprocessing Stage (Boundary Refinement of Cell Nuclei and False Findings Elimination)

After the segmentation stage, spurious regions such as blood cells or artifacts still existed in the image. It is essential to remove these false findings for better accuracy and robustness. A series of morphological operations (MO) were used to eliminate these false findings as well as to refine the boundaries of the segmented nuclei. A morphological opening method was applied to eliminate false findings that were smaller than a predetermined structuring element (SE). After performing this opening operation, the boundaries of cell nuclei often hold an irregular shape. A morphological closing operation was subsequently utilized to refine the shape or boundary of the cell nuclei.

An important consideration when applying MO is the size and shape of SE. SE identifies the pixels in the image being processed and also designates the neighborhood to be employed in the processing of each pixel. There are two parameters (shape and radius) of SE to be specified. In our algorithm, both opening and closing operations are achieved by using a disk shape with an SE radius of “n”. The SE radii “n” should be determined according to the size of the undesired objects to be removed [22]. However, it is difficult to set SE radii of “n” that can work well across all images in a dataset or across different nuclei within an image. The optimal radius should be closely related to the size of the false findings that need to be eliminated. Setting too large structuring element size oversimplifies the image, while using too small SE undersupplies the images (blood cells or noise remain). Hence, we applied a multiscale approach. This means that each image was processed with different SE radii. For the opening operation, we adapted the SE radii range to be n {7,8, .…., 15}, which corresponds approximately to the expected range of undesired objects in the pleural effusion cell nuclei. For the closing operation, a small SE (half the SE radii of the opening operation) size was adopted. The morphological opening and closing operations are mathematically formulated as follows:

(1) Segbi·SE=Segbi⊖SE⊕SE

(2) Segbi·SE=Segbi⊕SE⊖SE

where *Seg*_*bi*_ and *SE* denote the binary image and structuring element, respectively. ⊖ and ⊕ represent erosion and dilation, respectively. The visual results of this postprocessing are given in Figure 3(e).

### 3.4. Identification and Isolation of Overlapped Cell Nuclei

Most of the pleural effusion images in this study contain nuclei that overlap to different degrees. Isolation of overlapped cell nuclei is essential for optimal segmentation performance since the size and shape of cell nuclei need to be determined accurately for quantitative analysis. To the best of our knowledge, the isolation of overlapped cell nuclei in CPE images has only previously been addressed in our previous works mentioned above. In our previous studies, we employed watershed variants such as marker-controlled and distance transform watershed methods to split overlapped cell nuclei. Unfortunately, these methods suffered from oversplitting and did not perform well on images with a great deal of overlapped cells. Existing splitting methods for overlapped objects can be broadly grouped into watershed methods and contour concavity analysis. With these methods, the points to be separated are searched across all objects in an image, and it is then determined whether to split them or not. In contrast, we now propose the integration of shape analysis and concavity analysis to identify and split overlapped nuclei for better accuracy and robustness. The proposed method contains two substages: the identification of overlapped cell nuclei and their separation into individual ones, the details for which are given in Sections 3.4.1 and 3.4.2. Before any splitting process occurs, shape analysis is performed to judge whether nuclei are single or overlapped. If any overlapped nuclei are detected, a splitting process based on concavity analysis is carried out only on overlapped cell nuclei rather than on all nuclei in the image. This process can reduce computation time and also prevent oversplitting and undersplitting.

#### 3.4.1. Identification of Overlapped Cell Nuclei Using Shape-Based Analysis

**Figure 4:**
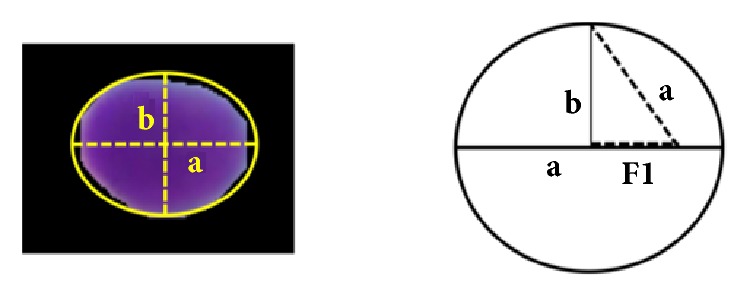
Formula of extracted features.

During this step, we aimed to develop a shape-based predetermination mechanism to identify the presence of overlapped cell nuclei. Identification of overlapped cell nuclei was performed in two consecutive steps: (i) key features were extracted from cell nuclei and (ii) the cell nuclei were classified into two classes: single nucleus or overlapped nuclei. It is our general observation that shape features are useful in helping to differentiate between individual and overlapped cell nuclei. Hence, we extracted a set of shape features, containing solidity, eccentricity, equivalent diameter, major axis length, and minor axis length. The formulation of shape-based features is explained and shown in Figure 4. The extracted key features given in Table 1 were utilized as input to SVM classifier [23] to classify and discriminate between single and overlapped cell nuclei. SVM classifier is a supervised learning mechanism that requires training with prelabeled training data. A trained SVM classifier was applied to identify overlapped cell nuclei in the image.

#### 3.4.2. Splitting Overlapped Cell Nuclei Using Concavity Analysis

**Figure 5:**
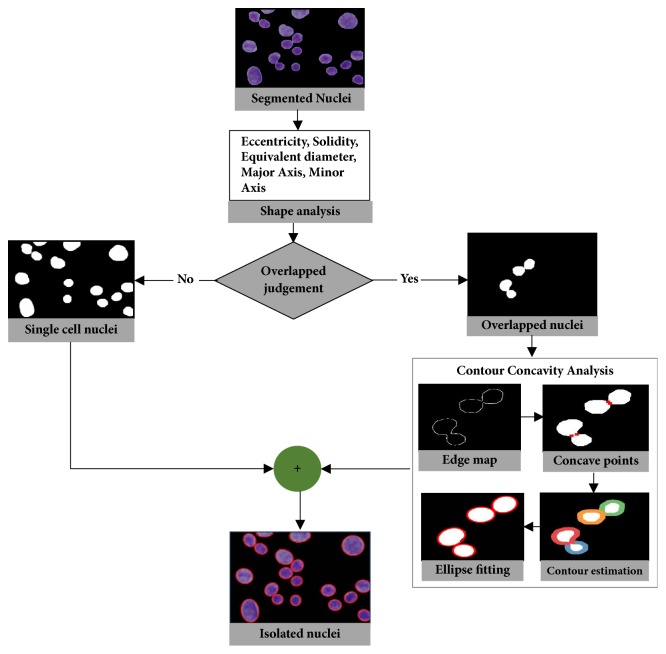
Visual demonstration of identification and splitting of overlapped cell nuclei.

When overlapped nuclei were identified via shape analysis, we separated the overlapped nuclei regions from the single nucleus regions. Then, contour concavity analysis (CCA), introduced in [24], was utilized to isolate the overlapped cell nuclei into individual ones. CCA includes contour evidence extraction and contour estimation. Contour evidence extraction involves two subprocesses: contour segmentation and grouping. In contour segmentation, canny edge method was utilized to extract the edge map. Then, curvature scale space (CSS) method based on curvature analysis was applied to detect the concave points representing the corner points of the object boundaries. Once the contour segments were obtained through the detection of concave points, the contour segments belonging to the same object were merged through a grouping process. The grouping process was performed using the properties of fitted ellipse. It groups contour segments of objects composed of an elliptical shape. When contour evidence was acquired, the contour estimation was carried out using a stable direct least square fitting method. The visual result of identification and isolation of overlapped cell nuclei is illustrated in Figure 5.

### 3.5. Features Extraction

After the cell nuclei were accurately delineated, feature extraction was established to extract the features that reflect the observation of cytologists. In the literature of cytology and histology image analysis, the dominant features for the diagnosis of malignancy used by cytologists are related to morphometric, colorimetric, and textural features [25–29]. In keeping with other cytological images, CPE images are also rich in various features like color, shape, and texture. In this study, 201 features related to the morphometric, colorimetric, and textural features were extracted and combined to obtain a robust, information-rich, and discerning feature set.

#### 3.5.1. Morphometric Features

There are certain differences in morphology between benign and cancer cell nuclei in CPE images. For instance, excessive growth of cell nuclei size and a significant variation of cell nuclei size in an image are suggestive of malignancy. Moreover, cell nuclei shape irregularities such as unsmooth nuclei margins occur in malignant cases. Thus, in this study, 14 morphometric features were extracted to evaluate nucleus size and shape irregularity. The description of these features is given in Table 2 and coded as F1-F14.

#### 3.5.2. Colorimetric Features

The usage of colorimetric features has tremendously increased in computer vision tasks due to their discriminative ability across different types of objects. Color provides useful information to determine malignancy. According to the cytological study, if any particular nuclei are affected by disease, the nucleus region changes in color. For instance, malignant cell nuclei become darker in color. In order to capture color features, means of R, G, B, H, S, and V components were extracted independently from RGB and HSV models. These features were coded in the range of F15 to F20.

#### 3.5.3. Textural Features

**Figure 6:**
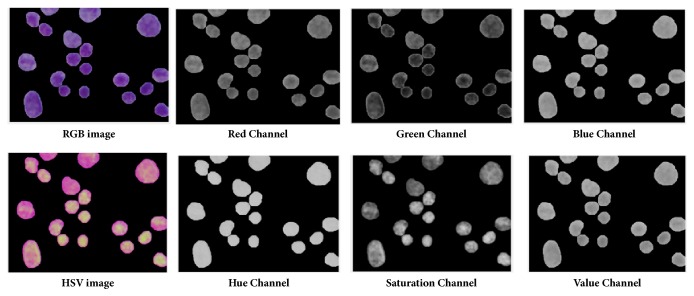
Individual color components of RGB and HSV color models in the segmented cell nuclei of CPE images.

In cytological pleural effusion images, malignant and cancer cell nuclei differ heavily in their distribution of color and chromatin. For instance, the frequent appearance of a distinct mass in a nucleus may be suggestive of malignancy. Texture features have been widely adopted in literature to exploit color and chromatin distribution. In this study, three statistical textural descriptors: first order statistics (FOS), gray level occurrence matrix (GLCM), and gray level run-length matrix (GLRLM) were employed to extract the textural features.

(*1) Color Component Based First Order Statistics (CCFOS)*. FOS describes the distribution of pixel intensities within a nucleus region [30]. In related literature, the combination of color and FOS features has achieved better accuracy compared to conventional FOS features [31, 32]. Thus, seven FOS features for seven color components (namely, gray, R, G, B, H, S, and V from RGB and HSV model) were extracted for each nucleus. The extracted features were named by color component based on FOS (CCFOS) and encoded from F21 to F69. The reason for extracting seven color components was to obtain FOS textures from the view of different color components. Different color components describe the different defined textures as given in Figure 6. The details of these extracted features are given in Table 3 and coded from F21 to F69.

(*2) GLCM and GLRLM*. FOS captures features only on individual pixels. It ignores the spatial relationship between neighboring pixels. In order to capture texture features that take into account the spatial relationship between neighboring pixels, GLCM [33, 34] and GLRLM [35] based higher order statistic features were considered. GLCM represents the distribution of cooccurring intensities in a nucleus at a specific given distance and orientation. When extracting GLCM features, it is required to define three parameters: distance (d) and orientations (***θ***) that determine the offset and angle between adjacent pixels, and the number of gray levels (NG) in the image. In this study, d and NG were set to 1 and 8, respectively. ***θ*** was adopted for four orientations 0°, 45°, 90°, 135° in order to take into account the rotation of the image. Thus, 22 GLCM features for four different orientations were extracted. GLRLM represents the length of homogeneous runs for each gray level in a definite direction. Similar to GLCM, GLRLM is constructed at four orientations and 8 gray levels. 11 GLRLM features in four different orientations (0°, 45°, 90°, 135°) were extracted. Tables 4 and 5 describe the lists of GLCM and GLRLM feature and their associated equations. Finally, a feature vector was generated by combining 14 features of form morphology and 6 color features and 181 textural features from CCFOS, GLCM, and GLRLM. The list of extracted features is given in Table 6. The class of each nucleus is labeled as either positive or negative class under the guidance of cytologists.

### 3.6. Feature Selection

The initial feature set contains 201 features related to morphometry, colorimetry, and texture. Directly utilizing all candidate features for classification may cause redundancy and irrelevancy. Redundancy can lengthen computation time. In turn, irrelevancy may cause poor predictive accuracy. To handle these problems, feature selection was performed in advance of classification. Feature selection is often applied in computer vision when many features get extracted. It improves the prediction performance and generalization capability and provides a faster and more cost-effective model. Feature selection is generally divided into two techniques: filter and wrapper [36]. In filter techniques, the features are chosen depending on their relevance ability with respect to the target. Filter methods are computationally fast and easy to implement. However, there is a possibility that the chosen features might contain redundant information since the selection process is carried out on the statistical measure of each feature. Unlike the filter approach, the wrapper approach depends on learning methods. It utilizes the estimated accuracy of the learning method as a performance measure to evaluate the usefulness of a feature. As an extension of the wrapper approach, the hybrid approach, which combines metaheuristics methods and supervised learning methods as integral components of feature selection, has been widely utilized in medical image analysis [37–39]. Experiments have found that hybrid methods are more efficient in finding optimal solutions compared to filter and wrapper methods. The main benefit of the hybrid methods is the ability to avoid being stuck in the local optima. In this study, a novel hybrid feature selection method based on hybridizing simulated annealing, one of the metaheuristics methods, with an artificial neural network, one of the popular machine learning methods, was developed to select the most relevant and informative features. The proposed method is known as a hybrid simulated annealing coupling artificial neural network (SA-ANN) feature selection. The details of SA-ANN are given in the subsection below.

#### 3.6.1. Hybrid SA-ANN Feature Selection

Simulated annealing is a global optimization algorithm that is inspired by the natural annealing process in metallurgy. It models the annealing process of heating material and then gradually cooling it by lowering the temperature at a controlled rate, thus minimizing system energy [40]. It is typically used to search for the global minimum in a high-dimensional data space. The main advantage of SA is that it allows up-hill moves in the iteration to avoid being stuck at a local minimum. SA has been widely used as a supervised or unsupervised feature subset selection method in data mining techniques, especially for microarray gene classification in biomedical data analysis [41–43]. Inspired by those works, in this study, we developed a novel hybrid feature selection method by hybridizing SA with an artificial neural network (ANN). ANN is a machine learning algorithm that mimics the structure of the biological brain. During feature selection via hybrid SA-ANN, the cost value of SA based search space was computed depending on the number of samples correctly predicted by ANN. Firstly, the random initial feature subsets were created. These subsets were assessed using a 3-layer ANN trained by a Levenberg-Marquardt (LM) backpropagation algorithm [44] containing a fixed number of hidden neurons. The features with the most minimal cost were initialized as the best feature set. At each iteration of SA, the neighboring subset was randomly generated by implementing a neighborhood function. Then, in a similar manner to the first stage, a 3-layer ANN trained by LM backpropagation algorithm was used to evaluate the cost of the neighboring subset. If the neighboring subset had a lower cost than the initial subset, we would then change the initial subset to its neighboring subset. Alternatively, if the neighboring subset had a higher cost, then the individual would move to that subset only if the acceptance probability condition was fulfilled. Otherwise, the individual remained in the initial subset. By accepting individuals that increase the cost, the algorithm avoids getting stuck by a local minimum in early iterations and explores globally for better solutions. As the algorithm progresses, the temperature is reduced causing individuals to converge towards the subset with a minimum cost and hence an optimal point. Hybrid SA-ANN feature selection can be summarized using the pseudocode in Algorithm 1, wherein feature set, MaxIt, Temp, and alpha are the candidate features, maximum numbers of iteration, initial temperature, and the temperature reduction rate, respectively. S_best is the output that represents the corresponding optimal feature set. The selected features in the optimal feature set were utilized for training and testing the classifier. The code implementation of proposed hybrid SA-ANN feature selection is based on the Matlab implementation available in [45] and modified as necessary.

### 3.7. Classification

**Figure 7:**
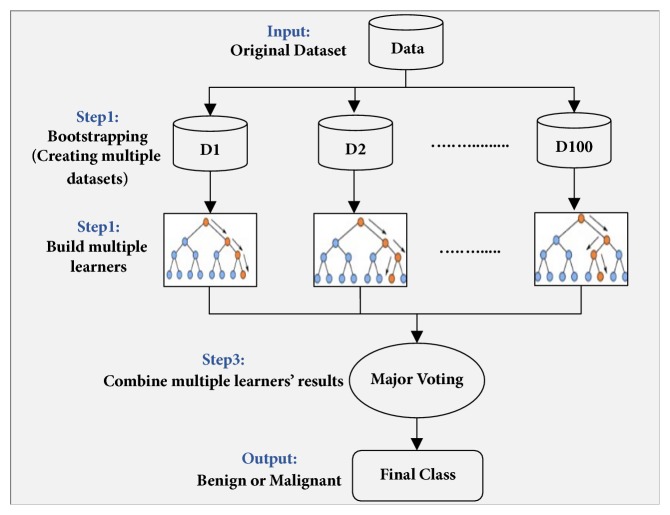
Block diagram of ensemble classifier of bagged decision trees (ECBDT) used in this study.

The selected features were utilized as input to the classifier to differentiate between benign and malignant cells. In cytology and histology image analysis, classification models revolve around Support Vector Machine (SVM) [26, 27], Naïve Bayes (NB) [27], artificial neural network (ANN) [28], K-nearest neighborhood (KNN) [8, 27], Logistic Regression (LR) [29], Linear Discriminant Analysis (LDA) [8], Decision Tree (DT) [46], and Ensemble Classifier (EC) [31]. The selection of a classification model for medical image analysis depends on the type and size of the dataset to be classified. Our dataset of cell nuclei was large and highly unbalanced wherein the class of cancer nuclei was limited while the class of benign nuclei was abundant. Ensemble classification has yielded preferable results for classification of skewed data [47, 48]. Thus, to deal with the unbalanced-data distribution, we adopted an ensemble classifier that employs bootstrap aggregation (bagging) decision trees and is termed as ECBDT [49, 50]. The core idea of using ECBDT was to develop multiple bootstrap data-samples and to build multiple base classifiers for each bootstrapped sample. One hundred decision trees were used as the base classifiers. The final prediction of ECBDT was obtained through major voting. The block diagram of the ECBDT classifier is depicted in Figure 7. The classifier was trained in 5-fold cross-validation.

## 4. Experiments

### 4.1. Experimental Setup

The proposed CAD system presented here was developed in a Matlab environment using a PC with Intel® Core i7,  CPU@3.40 GHz,  RAM@16.0 GB. The study was based on 125 cytology pleural effusion images containing around 10500 cells. The studied dataset was randomly partitioned into training and testing sets in an 80-20% ratio. 80% of the images were allocated to the training dataset to train the classifier and 20% to the testing dataset to validate the trained classifier. Training and testing datasets were disjointed (i.e., the same image was not assigned to represent both training and testing datasets). It is noteworthy that all the experiments carried out in this study are based on the same experimental setting and environment.

### 4.2. Experimental Results and Discussion

To obtain a comprehensive discussion, the experimental results are discussed in two phases. The first phase is the segmentation phase, which encompasses preprocessing, the segmentation of cell nuclei, postprocessing, and the isolation of cell nuclei. The second phase is the classification phase, which comprises feature extraction, feature selection, and classification.

#### 4.2.1. Segmentation Phase

**Figure 8:**
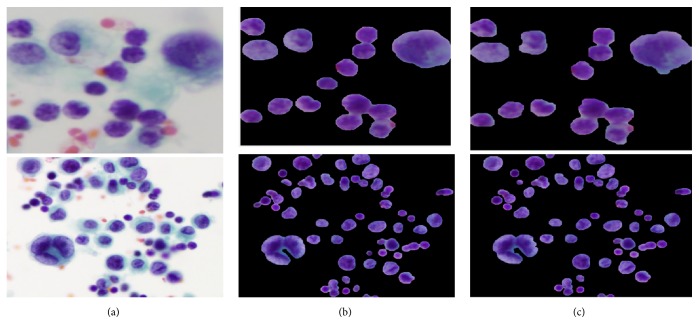
Comparison results of nuclei segmentation methods: (a) original image, (b) proposed method (SLIC + K-Means), and (c) K-Means clustering based segmentation.

Intensity adjustment and median filter methods were employed to enhance image contrast and suppress the noises, respectively. Then, a novel hybrid SLIC/K-Means segmentation method was developed to segment the cell nuclei from the entire image. In SLIC/K-Means, the SLIC method is firstly performed to presegment the image into the small compact superpixels. Then, K-Means clustering is carried out to cluster each superpixel into two groups by using the extracted features from superpixels. Features extracted over the uniform and compact SLIC superpixels tend to be more discriminative, helping K-Means to produce better segmentation. Good adherence to the image boundaries exhibited by SLIC superpixels results in smoother and more accurate segmentation. Utilizing K-Means clustering on superpixels can shorten computation because the number of superpixels is significantly lower than the number of pixels. It scales up linearly in computational cost and memory usage. The proposed segmentation method extracts cell nuclei at a lower computational cost and preserves the natural shape of the cell nuclei while achieving excellent segmentation results. In the hybrid SLIC/K-Means segmentation method, we need to specify two parameters: the number of superpixels for SLIC and the k clusters for K-Means. The desired number of superpixels was set to 500. According to our previous work, k was set at 2 because cell nuclei are segmented in a straightforward way when k is 2. False findings such as artifacts or blood cells may present obstacles to accurate segmentation. These undesired regions were filtered out with a series of morphological operations. Subsequently, the boundaries of cell nuclei were furthered refined. The visual results of the proposed SLIC/K-Means n and classical K-Means, supplemented by the same preprocessing and postprocessing approaches, are demonstrated in Figure 8. Compared to classical K-Means clustering based segmentation, the proposed method performs better in preserving the natural shape of the cell nuclei. Moreover, it is significantly faster than classical K-Means in computation, as given in Table 7.

**Figure 9:**
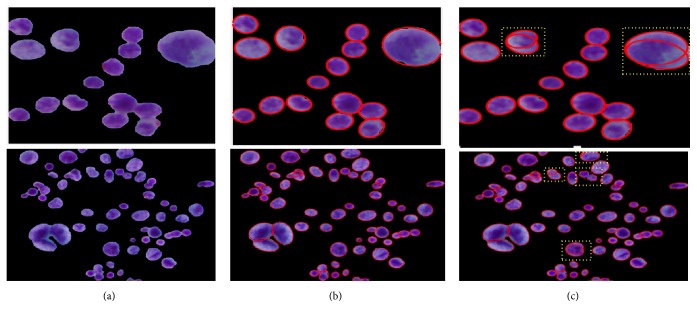
Comparison results of overlapped nuclei splitting methods: (a) segmented nuclei** (input)**, (b) proposed splitting method based on the combination of shape analysis and concavity analysis, and (c) contour concavity analysis** (note that the yellow rectangular box indicates the over- and undersplitting)**.

Almost all the images in the studied dataset possessed an overlapped cell nucleus to different degrees. Separating them into individual ones was hence essential. In almost all related literature, cell splitting is applied directly on the entire segmented image. This means that the splitting method is processed not only on overlapped regions but also on single cell nuclei regions. Such an attempt can lengthen computation time. In contrast, we propose a sequential combination of shape-based analysis and concavity analysis to identify overlapped areas and isolate them into individual ones. First, shape-based analysis was performed to determine the overlapped cell nuclei and separate them from single cell nuclei regions. Then, contour concavity analysis based splitting is applied only on the identified overlapped nuclei, rather than on all nuclei in the image. By identifying overlapped regions before applying the splitting method, one can not only prevent over- and undersplitting but also shorten computation time, as tabulated in Table 8. The visual results of splitting overlapped cell nuclei are illustrated in Figure 9.  Figure 9(a) shows the segmented nuclei image.  Figure 9(b) represents the resulting images from our proposed splitting methods (i.e., the combination of shape analysis and contour concavity analysis) and Figure 9(c) depicts the resulting images from classical contour concavity analysis. As shown in Figure 9(b), employing a splitting method only on the identified overlapped region can prevent the single cell nuclei from oversplitting and overlapped cell nuclei from undersplitting. This happens because the splitting method is focused solely on the overlapped area. The yellow shading box in Figure 9(c) is illustrated to highlight the over- and undersplitting which result from using the classical concavity analysis based splitting method.

#### 4.2.2. Classification Phase

**Figure 10:**
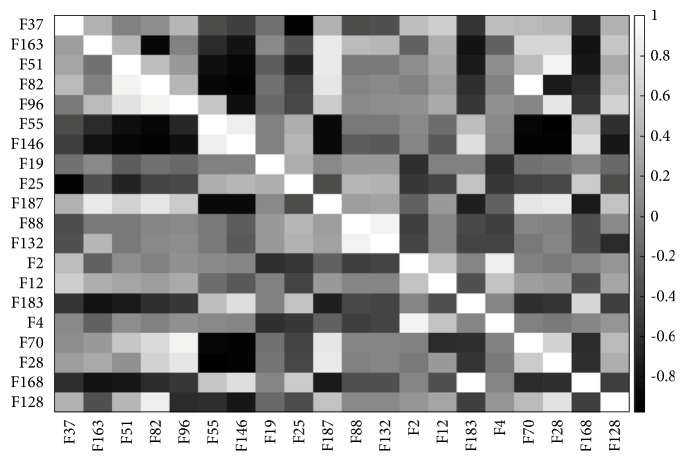
Correlation matrix for the selected features using hybrid SA-ANN feature selection (note that correlation =1 (white) means the highest correlation, -0 (black) no correlation).

Once the nuclei were accurately delineated, 201 features representing the morphometric, colorimetric, and textural features were extracted from each nucleus. In order to avoid redundancy and irrelevancy, hybrid SA-ANN feature selection was developed to choose the most discerning and informative features. Promising features that correctly map to the target are identified by supervised ANN and used in the annealing process. The SA-ANN algorithm was iterated 50 times with an initial temperature (temp=10) and temperature reduction rate (alpha=0.99). The algorithm was adapted to select a different desired number of features (nf) such as 15, 20, 25, 30, 35, and 40. Based on the experimental results obtained, it was deduced that selecting more than 20 features resulted in slightly decreased classification accuracy. Thus, the SA-ANN algorithm was fixed to select 20 features out of 201 features. The list of selected features and their correlation matrix are described in Table 9 and Figure 10, respectively. By analyzing the selected features, it was revealed that they included one or more representative features from each group of features given in Section 3.5. Among 20 selected features, 16 features were textural features. Thus, it is reasonable to conclude that textural features supply more diagnostic information than other features. Moreover, the correlation matrix demonstrates that proposed hybrid SA-ANN feature selection selected the most significant features with less redundant information. The selected features were used as input to the classification model to predict malignancy. Classification model choice depends on the size and the type of data to be predicted. Our data is highly skewed, wherein the cell nuclei, belonging to malignant (positive), were limited, and the cell nuclei belonging to benign (negative) were abundant. Thus, we adopted ensemble classification which provides preferable results to the classification of unbalanced data. As mentioned in Section 3.6, the dataset was firstly bootstrapped randomly, and 100 decision trees were used as the base classifiers to classify the bagged datasets. The final classification result was obtained through major voting. To evaluate classification performance, we compared the ground truth and classification results with respect to four performance metrics: sensitivity, specificity, F-score, and accuracy. These four performance measures are formulated in (3)-(8).

(3) Sensitivity=TruePositiveTruePositive+FalseNegative

(4) Specificity=TrueNegativeTrueNegative+FalsePositive

(5) Precision=TruePositiveTruePositive+FalsePositive

(6) Recall=Sensitivity

(7) F−score=2∗Precision∗RecallPrecision+Recall

(8) Accuracy=TruePositive+TrueNegativeTruePositive+FalsePositive+FalseNegative+TrueNegative

**Figure 11:**
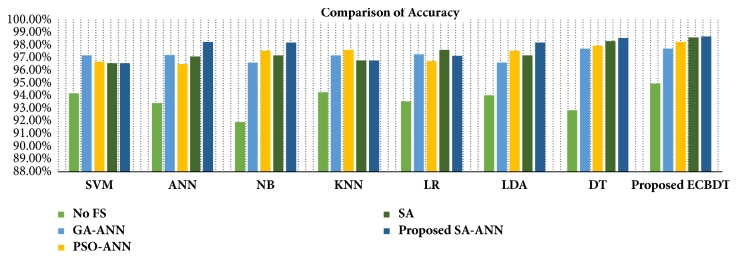
Comparison of accuracy using different pairs of feature selection methods and classifiers.

**Figure 12:**
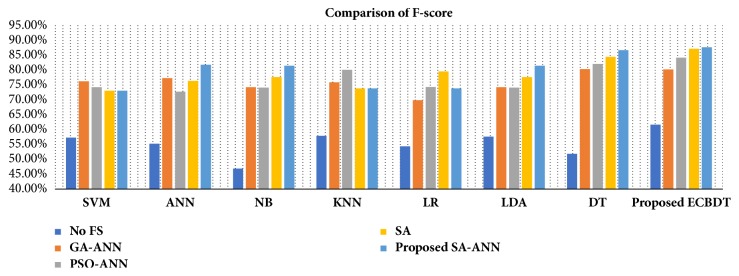
Comparison of F-score using different pairs of feature selection methods and classifiers.

**Figure 13:**
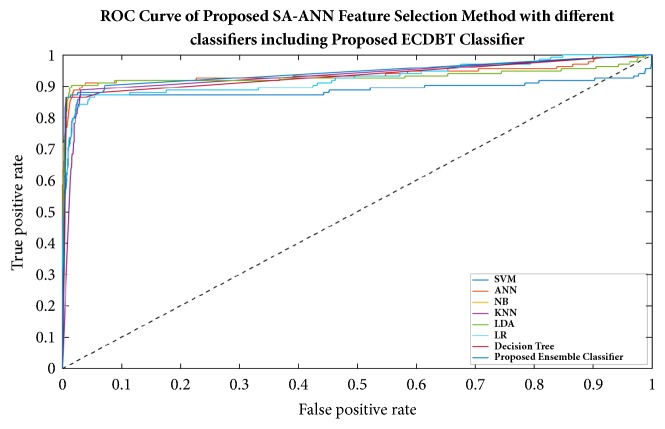
ROC curve for the performance of SA-ANN feature selection by blending with eight different classifiers.

**Figure 14:**
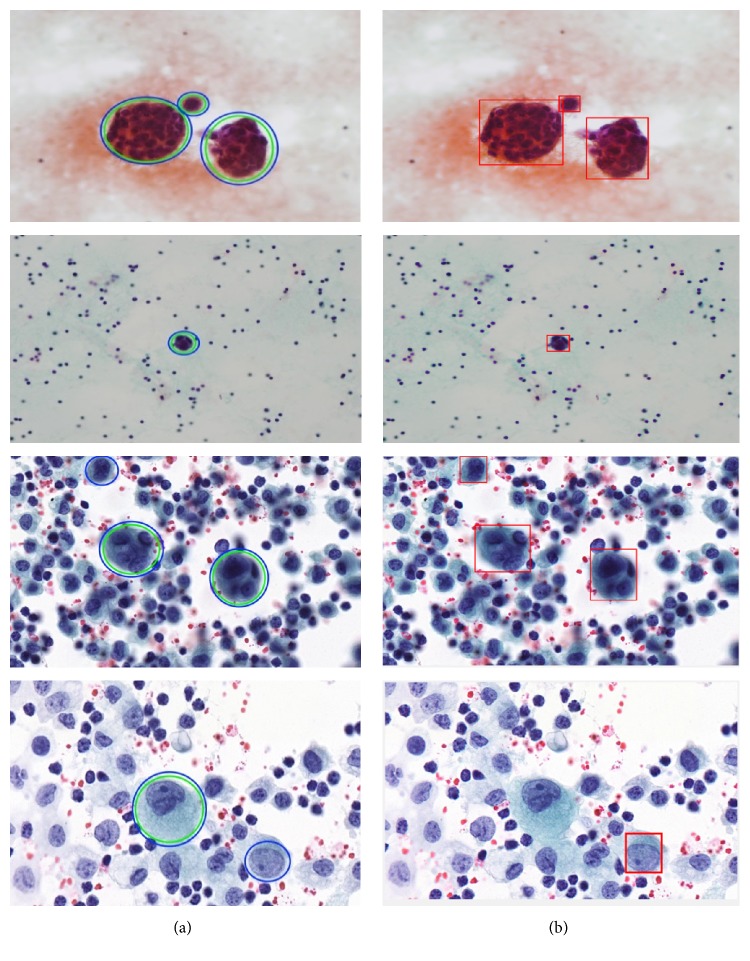
Visual demonstration of diagnostic results using the proposed CAD system to detect malignant cells in CPE images: (a) the original image with ground truth malignant cells annotated by two experts (blue and green circles represent the two experts) and (b) detected malignant cells through the proposed CAD system.

To make a fair and objective comparison, a common public dataset is required. By far, we are not aware of any common publicly available dataset. Also, the diagnosis schemes of CPE images in related literature are different from the proposed diagnosis scheme. Thus, we built our own experimental setup wherein the impact of using different feature selection methods and different classification models on classification performance was observed. In the first three experimental scenarios, we compared the classification accuracy achieved with and without features using the proposed classifier (i.e., ECBDT). In the first scenario, we compared the results between our proposed SA-ANN approach and an “all features” approach (i.e., without feature selection). Secondly, the result of the SA-ANN approach was compared with the results of the SA approach. In the third scenario, we established a comparison between the SA-ANN approach and other robust hybrid feature selection methods: PSO-ANN and GA-ANN approach. Furthermore, in the fourth experimental scenario, we employed seven alternative classifiers, namely, SVM [23], ANN [51], NB [52], KNN [53], LR [52], LDA [54], and DT [55] classifiers, and coupled them with the feature selection approaches. The result achieved by the proposed synergy between SA-ANN feature selection and ECBDT classification was compared with the results obtained through various pairings. Therefore, for each feature selection approach, the experimental results are presented with respect to four performance measures and eight classification models (including ECBDT). The results from four experimental scenarios are shown in Table 10. We clarify that hybrid SA-ANN coupling with an ECBDT classifier (shaded in bold) is our proposed method. As reported in Table 10, utilizing the feature selection methods (i.e., SA-ANN, SA, PSO-ANN, GA-ANN, or SA) provided better accuracy compared to the all features approaches (i.e., without feature selection) for all classifiers. The results also demonstrate that, with the exception of coupling with SVM, KNN, and LR classifiers, the proposed SA-ANN selection marginally improves accuracy compared to the SA based approach and yields better accuracy compared to PSO-ANN and GA-ANN approaches when coupling with ANN, NB, LD, DT, and proposed ECBDT classifiers. When coupling with an SVM classifier, the PSO-ANN approach yields better results compared to other selection approaches. Similarly, the GA-ANN approach yields better accuracy compared to other feature selection methods when coupling with KNN classifier. Likewise, the SA approach yields better accuracy compared to other feature selection methods when coupling with LR. The superior feature selection method for each classifier is shown in italic. It was observed that different classifiers perform differently for different selected features. However, regardless of the feature selection methods utilized, ECBDT (ensemble classifier) consistently provided better accuracy compared to other single classifiers. From the experimental results, it is inferred that the synergy of hybrid SA-ANN coupling with an ECBDT classifier outperformed other pairs of feature selection approaches and classification models described above in terms of classifying cells in CPE images. To get clear comparison results, we further plotted the comparison of accuracy and F-score as illustrated in Figures 11 and 12, respectively. Moreover, a Receiver Operating Characteristics (ROC) curve for different classifiers coupling with SA-ANN feature selection is depicted in Figure 13. It shows that the ROC curve of the proposed method is on the left upper corner and has higher classification rate stability when compared to other methods in the study. The visual results of detected malignant nuclei (both correct and failed cases) are depicted in Figure 14.  Figure 14(a) shows annotated malignant cell nuclei labeled by two experts in which blue and green represent the two experts.  Figure 14(b) describes the diagnostic results of the proposed CAD system wherein the red bounding boxes represent the detected malignant cells. Even though the proposed method yields promising results, there are still some failures especially when the malignant characteristics of a cell occur in the cytoplasm. Therefore, it remains for future work to detect for malignancy based on the combined analysis of cell nuclei and cytoplasm.

## 5. Conclusion

In this study, we presented a novel CAD system to detect cancer cells on CPE images. Firstly, intensity adjustment and median filter methods were employed to enhance image contrast and suppress noise, respectively. Then, the cell nuclei were extracted using a novel hybrid SLIC/K-Means segmentation method followed by postprocessing. Overlapped nuclei regions were then identified through shape-based analysis. Subsequently, concavity analysis was utilized to isolate the detected overlapping regions into individual ones. After the cell nuclei were accurately delineated, 201 features that comprise the morphometric, colorimetric, and textural features were extracted from each nucleus. A feature selection framework based on a hybrid SA-ANN was developed to select the most significant and informative features from the initial feature set containing those 201 features. The chosen features were used as input into ECBDT classifier to predict for malignancy. The proposed method can achieve 87.97% sensitivity, 99.40% specificity, 98.70% accuracy, and 87.80% F-score. The results achieved were compared with the results gained through an “all features”, SA, PSO-ANN, and GA-ANN approaches by coupling with eight different classifiers, namely, ECBDT, SVM, ANN, NB, KNN, LR, LDA, and DT. The comparison results demonstrated that the hybrid SA-ANN approach significantly improves accuracy compared to the “all features” approach for all classifiers. It marginally improves accuracy compared to the PSO-ANN, GA-ANN, and SA methods for most classifiers. Furthermore, the ECBDT classifier consistently improves classification performance compared to other individual classifiers: SVM, ANN, NB, KNN, LR, LDA, and DT. The proposed CAD system based on the synergy between SA-ANN feature selection and an ensemble classifier outperformed all other combinations conducted in this study. Nevertheless, there were still some failures, especially when the malignant characteristics of a cell occur in the cytoplasm. Hence, the future work of this research is to extend the combined analysis of cytoplasm and nuclei and further classify the detected malignant cells into different types, such as lung carcinoma, breast carcinoma, mesothelioma, and lymphoma. There is also a potential of adapting the proposed CAD system to the same kind of cytopathology images captured from other body fluid types such as the peritoneal, cerebrospinal, and synovial fluid.

## Figures and Tables

**Algorithm 1:**
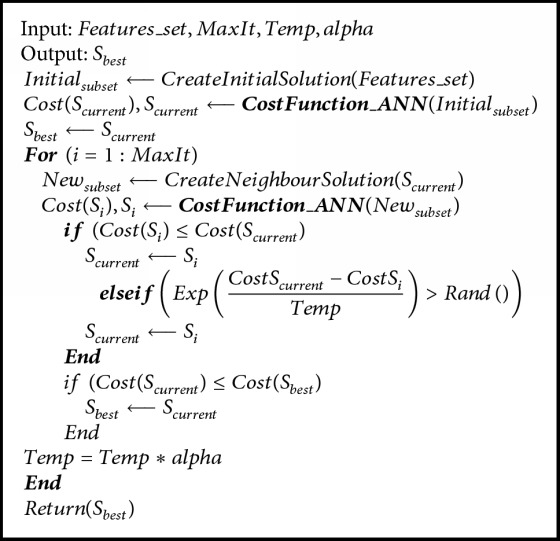
The main loop of hybrid SA-ANN based feature selection.

**Table 1:** Extracted shape based features and their equations.

**No.**	**Features**	**Formula**
(1)	Solidity	$\frac{Area}{ConvexArea}$

(2)	Eccentricity	$\frac{F1}{a}$

(3)	Equivalent Diameter	$\sqrt{\frac{4*Area}{pi}}$

(4)	Major axis length	2*∗a*

(5)	Minor axis Length	2*∗b*

**Table 2:** List of morphometric features and their associated equations.

Code	Feature Name	Equation
F1	Area	$\overset{n}{\underset{i=1}{\sum }}\overset{m}{\underset{j=1}{\sum }}S(i,j)$

F2	Perimeter	$Even\;\;count+\sqrt{2}\left(oddcount\right)$

F3	Roundness, circularity	$\frac{4\pi *Area}{Perimeter^{2}}$

F4	Solidity	$\frac{Area}{ConvexArea}$

F5	Equivalent circular diameter	$\sqrt{\frac{4\times Area}{\pi }}$

F6	Compactness	$\frac{Area}{Perimeter^{2}}$

F7	Eccentricity	$2*\frac{\left(\sqrt{{\left(ma/2\right)}^{2}-{\left(mi/2\right)}^{2}}\right)}{ma}$

F8	Diameter	$\frac{Perimeter}{2\pi }$

F9	Major axis length (*ma*)	$\sqrt{(x_{1}-x_{2}{)}^{2}-(y_{1}-y_{2}{)}^{2}\;\;}$

F10	Minor axis length (*mi*)	$\sqrt{(x_{2}-x_{1}{)}^{2}-(y_{2}-y_{1}{)}^{2}}$

F11	Elongation	ma/perimeter

F12	MaxIntensity	max⁡(*pixelValues*)

F13	MinIntensity	min(*pixelValues*)

F14	MeanIntensity	mean(*pixelValues*)

*S*(*i*, *j*) is the segmented image of rows *i* and columns*j*. *ma* and *mi* are the major axis and minor axis of the nucleus, respectively. *x*_1_, *y*_1_ and *x*_2_, *y*_2_ are the end points of the major axis and minor axis.

**Table 3:** List of CCFOS features and their associated equations.

Feature Name	Equation
Mean (*μ*)	∑_*i*=0_^*L*−1^*ip*(*i*)

Standard deviation(*σ*)	∑_*i*=0_^*L*−1^(*i* − *μ*)^2^∙*p*(*i*)

Smoothness	$1-\left(\frac{1}{1}+\sigma^{2}\right)$

Variance	∑_*i*=0_^*L*−1^(*i* − *μ*)^2^*p*(*i*)

Skewness	*σ* ^−3^∑_*i*=0_^*L*−1^(*i* − *μ*)^3^*p*(*i*)

Kurtosis	*σ* ^−4^∑_*i*=0_^*L*−1^(*i* − *μ*)^4^*p*(*i*) − 3

Energy	∑_*i*=0_^*L*−1^*p*(*i*)^2^

*p*(*i*) is the number of pixels with gray level *i*, and L represents the number of gray-level bins set for *p*.

**Table 4:** List of GLCM features and their associated equations.

**Features**	**Equations**
Autocorrelation	$\underset{i}{\sum }\underset{j}{\sum }\left(i∙j\right)p(i,j)$

Contrast	$\underset{i}{\sum }\underset{j}{\sum }\left|i-j{|}^{2}p(i,j\right)$

Correlation I	$\underset{i}{\sum }\underset{j}{\sum }\frac{\left(i-\mu_{x})(j-\mu_{y})p(i,j\right)}{\sigma_{x}\sigma_{y}}$

Correlation II	$\underset{i}{\sum }\underset{j}{\sum }\frac{\left(i∙j\right)p\left(i,j\right)-\mu_{x}\mu_{y}}{\sigma_{x}\sigma_{y}}$

Cluster Prominence	$\underset{i}{\sum }\underset{j}{\sum }{\left(i+j-\mu_{x}-\mu_{y}\right)}^{4}p(i,j)$

Cluster Shade	$\underset{i}{\sum }\underset{j}{\sum }{\left(i+j-\mu_{x}-\mu_{y}\right)}^{3}p(i,j)$

Dissimilarity	$\underset{i}{\sum }\underset{j}{\sum }\left|i-j\right|∙p(i,j)$

Energy	$\underset{i}{\sum }\underset{j}{\sum }p(i,j{)}^{2}$

Entropy	$-\underset{i}{\sum }\underset{j}{\sum }p(i,j)∙\log\left(p\left(i,j\right)\right)$

Homogeneity I	$\underset{i}{\sum }\underset{j}{\sum }\frac{p(i,j)}{1+|i-j|}$

Homogeneity II	$\underset{i}{\sum }\underset{j}{\sum }\frac{p(i,j)}{1+|i-j{|}^{2}}$

Maximum Probability	*max*_*i*,*j*_ *p*(*i*, *j*)

Sum of square	$\underset{i}{\sum }\underset{j}{\sum }\left(i-v{)}^{2}p(i,j\right)$

Sum average	$\overset{2L}{\underset{i=2}{\sum }}i∙p_{x+y}(i)$

Sum energy	$-\overset{2L}{\underset{}{\sum }}p_{x+y}(i)∙\log\left(p_{x+y}\left(i\right)\right)$

Sum variance	$\overset{2L}{\underset{i=2}{\sum }}\left(i-Sum\;\;engery{)}^{2}∙p_{x+y}(i\right)$

Difference variance	$\overset{L-1}{\underset{i=0}{\sum }}i^{2}∙p_{x-y}(i)$

Difference entropy	$-\overset{L-1}{\underset{i=0}{\sum }}p_{x-y}(i)∙log(p_{x-y}\left(i\right))$

Information measure of correlation I	$\frac{\left(-\sum_{i}^{}\sum_{j}^{}p\left(i,j\right)\cdot \log\left(p\left(i,j\right)\right)\right)-\left(-\sum_{i}^{}\sum_{j}^{}p\left(i,j\right)\cdot \log\left(p_{x}\left(i\right)p_{y}\left(j\right)\right)\right)}{\max\left(-\sum_{i}^{}p_{x}\left(i\right)\cdot \log\left(p_{x}\left(i\right)\right),-\sum_{i}^{}p_{y}\left(i\right)\cdot \log\left(p_{y}\left(i\right)\right)\right)}$

Information measure of correlation II	${\left(1-\exp\left[-2\left(\left(-\sum_{i}\sum_{j}p_{x}\left(i\right)p_{y}\left(j\right)\cdot \log(p_{x}\left(i\right)p_{y}\left(j\right)))-\left(-\sum_{i}\sum_{j}p\left(i,j\right)\cdot \log(p\left(i,j\right)\right)\right)\right)\right]\right)}^{1/2}$

Inverse Difference Normalized	$\underset{i}{\sum }\underset{j}{\sum }\frac{p(i,j)}{1+|i-j{|}^{2}/L}$

Inverse difference moment normalized	$\underset{i}{\sum }\underset{i}{\sum }\frac{p(i,j)}{1+(i-j{)}^{2}/L}$

*p*(*i*, *j*) is the (*i*, *j*)^*th*^ entry of the cooccurrence probability matrix, and *L* represents the number of gray levels used, while *μ*_*x*_, *μ*_*y*_ and *σ*_*x*_, *σ*_*y*_ are the mean and standard deviation of the *p*.

**Table 5:** List of GLRLM features and their associated equations.

**Features**	**Equations**
Short run emphasis (SRE)	$\frac{1}{n^{r}}\overset{G}{\underset{i=1}{\sum }}\overset{R}{\underset{j=1}{\sum }}\frac{g(i,j)}{j^{2}}$

Long run emphasis (LRE)	$\frac{1}{n^{r}}\overset{G}{\underset{i=1}{\sum }}\overset{R}{\underset{j=1}{\sum }}g\left(i,j\right)*j^{2}$

Low gray-level run emphasis (LGRE)	$\frac{1}{n^{r}}\overset{G}{\underset{i=1}{\sum }}\overset{R}{\underset{j=1}{\sum }}\frac{g(i,j)}{i^{2}}$

High gray-level run emphasis (HGRE)	$\frac{1}{n^{r}}\overset{G}{\underset{i=1}{\sum }}\overset{R}{\underset{j=1}{\sum }}g\left(i,j\right)*i^{2}$

Short run low gray-level emphasis (SRLGE)	$\frac{1}{n^{r}}\overset{G}{\underset{i=1}{\sum }}\overset{R}{\underset{j=1}{\sum }}\frac{g(i,j)}{i^{2}*j^{2}}$

Short run high gray-level emphasis (SRHGE)	$\frac{1}{n^{r}}\overset{G}{\underset{i=1}{\sum }}\overset{R}{\underset{j=1}{\sum }}\frac{g\left(i,j\right)*i^{2}}{j^{2}}$

Long run Low gray-level emphasis (LRLGE)	$\frac{1}{n^{r}}\overset{G}{\underset{i=1}{\sum }}\overset{R}{\underset{j=1}{\sum }}\frac{g\left(i,j\right)*j^{2}}{i^{2}}$

Long run high gray-level emphasis (LRHGE)	$\frac{1}{n^{r}}\overset{G}{\underset{i=1}{\sum }}\overset{R}{\underset{j=1}{\sum }}g\left(i,j\right)*i^{2}*j^{2}$

Gray level nonuniformity (GNU)	$\frac{1}{n^{r}}{\sum_{i=1}^{G}\left[\sum_{j=1}^{R}g(i,j)\right]}^{2}$

Run length nonuniformity (RNU)	$\frac{1}{n^{r}}{\sum_{j=1}^{MG}\left[\sum_{i=1}^{R}g(i,j)\right]}^{2}$

Run percentage (RP)	$\frac{n_{r}}{n_{p}}$

*g*(*i*, *j*) denotes the number of runs of pixels of gray level *i* and the run length *j*,*G* is the number of gray levels in the image, *R* is the number of different run lengths in the image, *n*_*r*_ is the total number of runs, and *n*_*p*_ is the number of pixels in the image.

**Table 6:** List of various features extracted from each nucleus.

Name of Feature sets	Number of Features	Ranges
Morphometric Features	14	F1-F14

Colorimetric Features	6	F15-F20

CCFOS (Textural Features)	49	F21-F69

GLCM (Textural Features)	88	F70-F157

GLRLM (Textural Features)	44	F158-201

Combined Feature Set	201	F1-F201

**Table 7:** Comparison of time complexity in segmentation methods using testing images.

Segmentation methods	Average processing time
Classical K Means	66.6 seconds
Proposed Method	5.8 seconds

**Table 8:** Comparison of time complexity in splitting methods using testing images.

Splitting methods	Average processing time
Concavity analysis	10.2 seconds
Proposed method	6.8 seconds

**Table 9:** Description of selected features through hybrid SA-ANN feature selection.

**No.**	**Feature Code**	**Feature Name**	**Feature Set**
(1)	F37	Smoothness of B component	CCFOS

(2)	F163	Short run high gray-level emphasis	GLRLM0

(3)	F 51	Smoothness of S component	CCFOS

(4)	F 82	Sum of square	GLCM0

(5)	F 96	Cluster Prominence	GLCM45

(6)	F 55	Energy of S component	CCFOS

(7)	F 146	Homogeneity II	GLCM 135

(8)	F 19	Mean color of S component	Colorimetric

(9)	F 25	Skewness of R component	CCFOS

(10)	F 187	Long run high gray-level emphasis	GLRLM 90

(11)	F 88	Information Measure of Correlation	GLCM0

(12)	F 132	Difference Entropy	GLCM 90

(13)	F 2	Perimeter	Morphometric

(14)	F 12	MaxIntensity	Morphometric

(15)	F 183	High gray-level run emphasis	GLRLM 90

(16)	F4	Solidity	Morphometric

(17)	F 70	Autocorrelation	GLCM 0

(18)	F 28	Mean from G component	CCFOS

(19)	F 168	Run percentage	GLRLM0

(20)	F 128	Sum Entropy	GLCM0

**Table 10:** Comparison of classification performance achieved by different synergy between feature selection methods and classification models.

**Feature Selection (FS)**	**Performance Metrics**	**Classifiers**							
		**SVM**	**ANN**	**NB**	**KNN**	**LR**	**LDA**	**DT**	**Proposed ECBDT**
All features (No FS)	Sensitivity	72.18%	75.19%	66.17%	72.93%	71.43%	75.19%	71.43%	74.48%
	Specificity	95.47%	94.48%	93.41%	95.51%	94.82%	95.12%	94.10%	96.11%
	F-score	57.31%	55.25%	46.93%	57.91%	54.44%	57.64%	51.91%	61.73%
	Accuracy	94.21%	93.44%	91.95%	94.29%	93.57%	94.05%	92.88%	94.98%

PSO-ANN	Sensitivity	*73.65*%	70.91%	69.16%	74.29%	69.23%	69.16%	71.83%	76.47%
	Specificity	*96.64*%	96.11%	95.67%	96.72%	96.11%	95.67%	96.32%	97.09%
	F-score	*76.29*%	77.33%	74.30%	75.96%	69.96%	74.30%	80.42%	80.28%
	Accuracy	*97.21*%	97.25%	96.64%	97.21%	97.29%	96.64%	97.73%	97.73%

GA-ANN	Sensitivity	87.97%	86.47%	64.66%	*87.97*%	87.22%	64.66%	86.47%	86.47%
	Specificity	97.22%	97.09%	99.44%	*98.20*%	97.31%	99.44%	98.63%	98.93%
	F-score	74.29%	72.78%	74.14%	*80.14*%	74.36%	74.14%	82.14%	84.25%
	Accuracy	96.72%	96.52%	97.57%	*97.65*%	96.76%	97.57%	97.98%	98.26%

SA	Sensitivity	85.71%	86.47%	90.23%	84.21%	*84.96*%	90.23%	84.21%	87.22%
	Specificity	97.22%	97.73%	97.60%	97.52%	*98.37*%	97.60%	99.14%	99.27%
	F-score	73.08%	76.41%	77.67%	73.93%	*79.58*%	77.67%	84.53%	87.22%
	Accuracy	96.60%	97.13%	97.21%	96.80%	*97.65*%	97.21%	98.34%	98.62%

Proposed SA-ANN	Sensitivity	85.71%	*72.93*%	*72.93*%	84.21%	79.70%	*72.93*%	*86.47*%	**87.97**%
	Specificity	97.22%	*99.70*%	*99.66*%	97.52%	98.16%	*99.66*%	*99.27*%	**99.40**%
	F-score	73.08%	*81.86*%	*81.51*%	73.93%	75.18%	*81.51*%	*86.79*%	**87.79**%
	Accuracy	96.60%	*98.26*%	*98.22*%	96.80%	97.17%	*98.22*%	*98.58*%	**98.70**%

## Data Availability

The data used to support the findings of this study are available from the corresponding author upon request.
